# Biologically Active Peptides from Venoms: Applications in Antibiotic Resistance, Cancer, and Beyond

**DOI:** 10.3390/ijms232315437

**Published:** 2022-12-06

**Authors:** Lucía Ageitos, Marcelo D. T. Torres, Cesar de la Fuente-Nunez

**Affiliations:** 1 Machine Biology Group, Departments of Psychiatry and Microbiology, Institute for Biomedical Informatics, Institute for Translational Medicine and Therapeutics, Perelman School of Medicine, University of Pennsylvania, Philadelphia, PA 19104, USA; 2 Departments of Bioengineering and Chemical and Biomolecular Engineering, School of Engineering and Applied Science, University of Pennsylvania, Philadelphia, PA 19104, USA; 3 Penn Institute for Computational Science, University of Pennsylvania, Philadelphia, PA 19104, USA

**Keywords:** antimicrobial peptides, venom, cancer, South America, neurotoxins

## Abstract

Peptides are potential therapeutic alternatives against global diseases, such as antimicrobial-resistant infections and cancer. Venoms are a rich source of bioactive peptides that have evolved over time to act on specific targets of the prey. Peptides are one of the main components responsible for the biological activity and toxicity of venoms. South American organisms such as scorpions, snakes, and spiders are important producers of a myriad of peptides with different biological activities. In this review, we report the main venom-derived peptide families produced from South American organisms and their corresponding activities and biological targets.

## 1. Introduction—Drug Discovery in Venoms

Venoms are evolved complex mixtures of toxins that take part in the defensive and predation processes of the producer organism [1,2]. These toxins typically display toxicity against neuronal, red blood, and other mammalian cells, but also therapeutic biological activities such as antimicrobial, antitumoral, or analgesic properties [3]. Indeed, venoms can contain up to 500 biologically active compounds and are considered a rich source of potential drugs with novel molecular scaffolds [3,4,5]. Although it is estimated that up to 50 million different natural products exist in venoms, only 0.01% of such molecules have been identified and characterized [3]. The main reasons for this limitation are the challenging isolation and purification processes involved, and the low amount of these molecules produced by the venomous animals [5]. Recent advances in bioanalytical technologies, genomics, transcriptomics, and computational biology have enabled obtaining new insights into the structure and function of the venom content [5,6,7,8,9,10,11,12]. Such studies revealed, for example, that venoms are mainly composed of a heterogenous mixture of inorganic salts, low-molecular-weight organic molecules, peptides (2–10 KDa), and enzymes (>10 KDa), even though the pharmacology and complexity of their content differ substantially between venomous animals [2,4]. In recent years, there has been an increase in the number of reports describing therapeutic peptides isolated from venoms with potent activity in preclinical animal models [13]. Multiple venomous organisms of interest, such as insects, arthropods, and reptiles, can be found in different parts of South America. In this review, we highlight peptides with interesting biological features isolated from venoms of organisms originally from South America.

## 2. Venom-Derived Peptides 

Available treatments for drug-resistant infections and cancer have side effects and have lost effectiveness due to the emergence of evolved resistant bacteria and tumors [14,15]. In addition, the drug development pipeline in these areas, particularly antimicrobial-resistant (AMR) infections, has not yielded truly novel structural candidates in decades [16]. AMR infections are responsible for 700,000 deaths annually [17], and cancer is the second leading cause of death in our society, reaching a ratio of 1 out of 6 deaths and with an estimated 27.5 million cases and 16.3 million deaths by 2040 [18]. Venom-derived peptides have been exploited as novel potential medicines for the treatment of AMR infections and cancer. This class of peptides comprises short biopolymers formed by amino acid residues that naturally evolved over time to reach specific targets in the prey, such as G protein-coupled receptors, ion channels, or enzymes, without suffering proteolytic cleavage [4,5]. Venom-derived peptides are also known for displaying therapeutic properties, including bacteria and cancer-targeting capabilities [19,20]. Their structural characteristics allow them to interact selectively with cancer and bacterial cell membranes through electrostatic interactions followed by hydrophobic interactions with the lipid bilayer [21]. The activity displayed by these compounds correlates with their physicochemical properties, including net charge, hydrophobicity, and solvent accessibility, which in turn dictate their mechanisms of action, selectivity, and specificity towards their targets [12]. Even though many venoms contain antimicrobial peptides (AMPs), other families of biologically active peptides can be also found in venoms [22], such as ICK peptides and α- and β-toxins.

### 2.1. Snake Venoms

Snake venoms are known for their toxicity, causing over 80,000 human deaths annually [23,24]. Components from these venoms have evolved to interact with specific mammalian proteins [3], and with the muscles, brain, nervous and cardiovascular systems of their prey, exhibiting high cyto-, neuro-, and hemotoxicity [25]. Snake bites often lead to punctured wounds that rarely get infected, despite the presence of microbes in the oral cavity of the snake [26,27]. This characteristic extended throughout different snake families (e.g., vip rippers and rattlesnakes), and has been at least partially explained by the presence of AMPs [26]. AMPs are commonly produced as part of host defense mechanisms, are also referred to as endogenous host defense peptides (HDPs), and can be found in the venom of multiple organisms [28]. These peptides are generally short cationic peptides containing fewer than 100 amino acid residues and hydrophobic and amphipathic properties [21].

**Figure 1:**
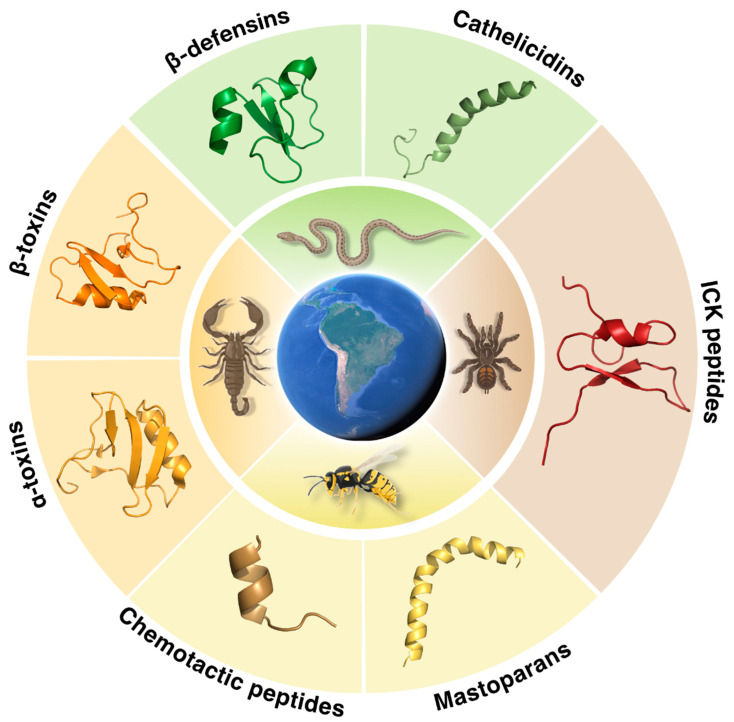
Families of venom-derived peptides from South American organisms. Schematic representation of the venom producers from South America and main peptide families found in their venoms, such as β-defensins (e.g., crotamine), cathelicidins (e.g., crotalicidine), ICK peptides (e.g., psalmotoxin 1), α-toxins (e.g., Ts1), β-toxins (e.g., Ts3), mastoparans, and chemotactic peptides (e.g., polybia-CP). This figure was created with BioRender.com.

Snake venom AMPs (SV-AMPs) mainly belong to the defensin-like and cathelicidin peptide families (Figure 1). The best example organism is the rattlesnake *Crotalus durissus terrificus,* a producer of both peptide families. Defensins are endogenous cationic peptides that contain from six to eight conserved cysteine residues that stabilize the β-sheet structure of the peptide by forming three disulfide bridges [25,29]. There are three types of defensins (α, β, and θ), which differ in the distance between cysteine residues within the sequence [29,30]. Specifically, β-defensins are interesting antimicrobial peptides composed of 18–45 amino acid residues [31]. These peptides take part in the defense barrier of vertebrates and display broad-spectrum activities against bacteria, fungi, and viruses [32]. Crotamine, from the rattlesnake *Crotalus durissus terrificus,* is a 42-amino acid residue β-defensin-like myoneurotoxin described as an antimicrobial and cell-penetrating peptide (CPP) (Figure 1 and Table 1). Even though this peptide presented cytotoxicity in vivo (2.5 mg Kg^−1^), leading to limb paralysis and necrosis, and in vitro toxicity against muscle cells (10 μg mL^−1^) [25,32], it also displayed significant antimicrobial and anticancer activities. Crotamine was one of the first AMPs that was also described as a CPP with selective antifungal activity [25,33]. The CPP properties of crotamine have been explored to selectively target cancer cells by interacting with the mitochondria and lysosomes without any observed side effect or alteration to normal cells [25,33,34]. 

Cathelicidins (CATHs) are likely the larger family of SV-AMPs and SV-CPPs peptides worldwide. Indeed, 25 SV-CATHs have been identified to date [27]. CATHs are released upon proteolytic cleavage of a precursor that encodes the bioactive peptide in a hypervariable C-domain, hence it is possible to find different types of peptides inside this family. Notwithstanding, the majority of CATHs are short and linear peptides of 25–35 amino acid residues with an amphipathic α-helical structure [27,48,49,50]. In addition to their known antimicrobial and anticancer activities, CATHs also present immunomodulatory properties by acting on biological processes such as wound healing, angiogenesis, or the inhibition of apoptosis, among others [48,51]. South American pit vipers produce cathelicidin-related antimicrobial peptides (CRAMPs) named vipericidins. This family of peptides possesses an anionic moiety followed by a Lys-/Arg-rich and amphipathic domain [35]. Vipericidins are usually active at low micromolar concentrations (0.05–3.8 μmol L^−1^) against AMR infections caused by Gram-negative bacteria. In contrast, vipericidins are only active against Gram-positive bacteria at concentrations ranging from 16 to 128 μmol L^−1^. Toxicity studies showed that these peptides led to 50% hemolysis at millimolar concentrations, similar to the human defensin LL-37, allowing a selectivity window of 2-log for targeting bacteria versus eukaryotic cells [35,50]. *Crotalus durissus terrificus* venom also contains crotalicidin, a 34 amino acid residues vipericidin that, in hydrophobic environment, structures into an α-helix at the N-terminal while keeping random coil conformation at the C-terminal of the peptide (Figure 1, Table 1) [27]. Crotalicidin showed broad antibacterial activity against Gram-negative and Gram-positive bacteria (MIC < 10 μmol L^−1^), potent anticancer activity against leukemia cells (IC_50_ < 5 μmol L^−1^), antiparasitic activity and immunomodulatory activities by promoting autoinflammation with low toxicity levels (10% hemolysis at 25 μmol L^−1^) [27,35]. These activities are derived from the ability of this peptide to interact and disrupt the targeted bacterial membranes and to display membranolytic effects while interfering with intracellular pathways in cancer cells [27]. 

### 2.2. Spider Venoms

Spiders produce venoms, which are lethal complex cocktails of toxins (neurotoxins, enzymes, proteins, biologically active peptides, nucleotides, salts, amino acids, and neurotransmitters) that allow them to overcome preys that can be considerably larger [52]. Some of these venoms are so potent that even small amounts can be lethal to humans. Up to 1000 peptides (2–10 kDa) can be found in a crude venom sample, making them the main component of spider venoms [53]. It is estimated that there are approximately 12 million peptides from spider venoms, most of them still uncharacterized. As for snakes, peptides are typically produced to clean the biological conducts where the venom is transported from the gland to the tip of the spider fangs to block potential infections [54]. Peptides bearing an inhibitory cysteine knot (ICK) in their structure, known as ICK peptides or knottins, are the most abundant class of peptides in spider venoms and are the ones responsible for the neurotoxicity displayed by these venoms (Figure 1) [52,55]. These peptides are small (commonly from 3 to 10 KDa) and present from 6 to 14 cysteine residues that create a knot-like structure composed of antiparallel β-sheets with a ring disposition, made by two disulfide bonds, which are crossed by a third disulfide bond [52,55]. This motif contributes to the rigidity and stability of the peptides against thermal and enzymatic degradation and helps maintain their active conformation so they can interact with their target [52]. Ion channels (e.g., voltage-dependent sodium (Na_v_), potassium (K_v_), and calcium (Ca_v_) channels) and neuroreceptors are the main targets of ICK peptides, leading to not only antimicrobial activity but also antiarrhythmic, analgesic, antiparasitic, cytolytic, hemolytic, and enzyme inhibitory activities [56].

*Phoneutria nigriventer* is one of the most important sources of ICK peptides from South America [36]. This spider, also known as “armed” spider, is an extremely aggressive and dangerous spider that can be found throughout South America [57]. The bite from *P. nigriventer* causes severe and irradiating pain along with toxic symptoms such as convulsions, arrhythmias, spastic paralysis, or priapism [58]. These symptoms are generated by the production of neurotoxic ICK peptides that cross the blood–brain barrier and interact with ionic channels and different receptors from the nervous system [37,57,58]. The knottins display a wide range of biological activities caused by their different mechanisms of action, such as inactivation of Na^+^ channels or blockage of voltage-gated calcium channels (VGCCs) [58]. To date, 41 neurotoxins from *P. nigriventer*, divided into 5 fractions (PhTx-1 to 5), have been identified (Table 1) [37]. Intracerebral administration of the different fractions (PhTx-1 to 5) in vivo showed toxic symptomatology in all cases, including excitation, spastic paralysis, progressive flaccid paralysis, hyperactivity, and smoothing of the muscles [37]. All the bioactive peptides found in these fractions are cysteine-rich peptides with lengths that vary from 34 to 78 amino acid residues [37]. Further biological studies on each of the peptides found in the venom of *P. nigriventer* showed their therapeutic potential primarily due to their ability to interact with ion channels and opioid receptors [37]. Most of these peptides show promising antinociceptive effects in different pain models (e.g., chronic pain, cancer-related pain, postoperative pain syndrome, or treatment-related pain) [37]. Indeed, they exhibited similar or more favorable antinociceptive effects than opioids, specifically morphine, and the well-known toxin ω-conotoxin MVIIA. These peptides generated no side effects and were able to revert tolerance to opioids in mice [37]. However, in addition to their antinociceptive properties, these peptides showed neuroprotective properties, insecticidal activity, and potential in the treatment of erectile dysfunction treatment, among others [37].

Another important ICK venom-derived peptide to mention is Psalmotoxin 1, also known as PcTx1, isolated from *Psalmopoeus cambridgei*, a tarantula originally from the West Indies (Figure 1, Table 1). PcTx1 is composed of 40 amino acid residues and was the first compound to be described as an inhibitor of the acid-sensing ion channels (AISCs) that are extensively expressed throughout the nervous system [38,39]. Study of the therapeutic role of PcTx1 in different in vivo pain models, such as acute and neuropathic pain, showed similar or even better antinociceptive effects than morphine and reverted thermal hyperalgesia and tactile allodynia without side effects. This peptide also showed inhibition of the AISCs expressed in glioblastoma multiforme (GBM) (IC_50_ of 36 pmol L^−1^), while not affecting normal cells, thus constituting a promising candidate for the treatment of cancer [39].

Spiders from the genus *Loxosceles* cause the necrotic and systemic disease known as loxoscelism. Although some of the *Loxosceles* genus members, such as *Loxosceles intermedia*, produce ICK peptides, others such as *Loxosceles gaucho* present biologically active peptides that do not belong to the family of ICK peptides. The study of the composition of *L. gaucho* venom showed the presence of U1-SCRTX-Lg1a, a hydrophobic anionic antimicrobial peptide (AAMP) composed of 16 amino acid residues (Table 1). Contrary to broad-spectrum AMPs, this peptide was selective against Gram-negative bacteria (MIC 1.5–4.6 μmol L^−1^), displaying even increased activity compared to the potent peptide gomesin isolated from the spider *Acanthoscurria gomesiana* (MIC < 6.5 μmol L^−1^). U1-SCRTX-Lg1a was not hemolytic (approximately 0.15% hemolysis at 137 μM) or cytotoxic against HeLa cells at any of the concentrations tested (i.e., 0.88–112.74 μmol L^−1^) [40]. These data indicate that the AMP U1-SCRTX-Lg1a, considered an important component of the innate immunity of the spider *L. gaucho*, interacts with an intramolecular target after translocating into the bacterial cell. The peptide acts through non-membranolytic mechanisms by creating cationic salt bridges between the negatively charged components of the bacterial cell membrane and metallic chelates in its aspartic and glutamic acids [40].

### 2.3. Scorpion Venoms

Scorpion venoms are rich arsenals of biological compounds that comprise mainly small neurotoxic peptides along with minor components such as sugars, salts, the neurotransmitter serotonin, and protease inhibitors [41,59,60]. Scorpion venoms display toxicity against both vertebrate and invertebrate organisms [59]. The neurotoxicity displayed is related to the peptide content present in their venom and, more specifically, due to the production of α- and β-toxins (Figure 1). Both families of peptides are composed of 58–76 amino acid residues, and their structure is stabilized by disulfide bridges [59]. α- and β-toxins target and interact with the Na_V_ channels present in the membrane of excitable and non-excitable cells, affecting their ion flux [41,59,60]. Specifically, α-toxins extend the depolarization time of Na_V_ channels leading to extended potential action in nerves and muscles [59,60] whereas β-toxins change the Na_V_ channel activation threshold leading to hyperpolarized membrane voltages [41]. Scorpions from the genus *Tityus* are important producers of these toxins and, in Brazil alone, 22 different scorpions from this genus have been identified [59].

*Tityus serrulatus* has been rated as the most dangerous scorpion in Brazil [41]. Its envenomation can induce hyperglycemia, glycogenolysis, leukocytes, or even pulmonary edema, which can lead to death [41]. *Tityus serrulatus* venom (Tsv) has been extensively studied over the years due to its rich peptide content, namely small neurotoxins bearing disulfide bonds able to interact with ionic channels, and linear peptides with specific activity on receptors. Insights into its peptide content led to the characterization of 14 peptides belonging to different families such as α- and β-toxins or K^+^ channel neurotoxins (Table 1) [41]. One of the most important toxins found in this venom, Ts1, is a β-like neurotoxin with high affinity for mammalian Na_V_ channels, which showed competition with AahIT, the most potent excitatory insect-selective toxin (Figure 1, Table 1) [60]. Structurally, Ts1 is composed of three antiparallel β-strands and an α-helix bonded by disulfide bridges with a significantly different orientation compared to that of α-toxins [41]. On the other hand, Ts3 is the only α-neurotoxin found in this venom (Figure 1, Table 1). This peptide, composed of 62 amino acid residues, inhibits the inactivation of Na_V_ channels, favors the release of neurotransmitters, and depolarizes nitrergic fibers to induce smooth muscle relaxation [41].

### 2.4. Wasp Venoms

Peptides comprise up to 70% of dried wasp venoms, while the rest of the content contains other biologically active compounds such as biogenic amines, polyamine toxins, enzymes, and allergens [46,61]. Mastoparans and chemotactic peptides are the main peptides produced in wasp venoms, although it is also possible to find neurotoxic peptides and kinins (Figure 1) [61]. Mastoparans are short cationic peptides with an amidated C-terminal leucyl residue, two to four lysine residues, and no cysteine residues. This family of peptides is involved in the inflammation, hemolysis, and cell membrane lysis that occur upon envenomation. In water, mastoparans present a random coiled structure, and upon contact with the target membrane, their structure adopts an amphiphilic α-helix conformation in which the hydrophobic and hydrophilic residues are distributed in opposite faces of the helix (Figure 1) [46,61,62]. This structural transition leads to the creation of pores and destabilization of bacterial membranes through different membrane-related mechanisms of action, such as the carpet model and the toroidal pore formation [47,61]. Their ability to translocate or permeabilize membranes, also including that of the mitochondria, make these peptides versatile therapeutic alternatives for the treatment of bacterial, fungal, viral infections, and even cancer [46]. However, mastoparans also target erythrocytes and mast cells, showing unwanted hemolytic and mast cell degranulation activities [61]. The latter activity can be partially explained by the ability of mastoparans to mimic G protein-coupled receptors (GPCRs) allowing the activation of these proteins and leading to the production of pores in lipidic membranes [46,61,62]. For instance, Polybia-MPII, a mastoparan peptide isolated from *Pseudopolybia vespiceps Testacea*, which is an important source of biologically active peptides [46,63], presents potent antimicrobial activity against Gram-positive (MIC = 2-5 μmol L^−1^) and modest activity against Gram-negative (MIC = 5-38 μmol L^−1^) bacteria. However, this peptide is hemolytic at an ED_50_ of 5 × 10 ^−5^ mol L^−1^ (Table 1) [47,64].

Chemotactic peptides are composed of 12 or 13 amino acid residues, are mainly hydrophobic, and possess a single basic residue. These peptides typically cause hemolysis and reduce mast cell degranulation [47]. Polybia-CP, for example, is a chemotactic peptide composed of 12 amino acid residues isolated from the venom of the social wasp *Polybia paulista* (Figure 1, Table 1). This peptide reduced mast cell degranulation (10^−5^ mol L^−1^) and exhibited low hemolytic activity [47] and antichagasic activity [63]. In a comprehensive study, the structure of polybia-CP was elucidated, and amino acid substitutions throughout the peptide using alanine residues revealed insights into the specific role of each of these residues on biological function (Figure 1) [65]. The hydrophobic face of the peptide’s helical structure was crucial for its activity, while substitutions made within its hydrophilic face did not affect the helicity nor the antimicrobial activity of the peptide. Further studies with the same peptide highlighted the importance of the position of the positively charged residues within the hydrophilic face of the peptide, which led to increased helical content and antimicrobial activity. This structure-function-guided design approach led to an optimized hit, [Lys]^7^-Polybia-CP, with improved structural features, such as net positive charge and helicity, in addition to lower toxicity and increased antimicrobial in vivo activity at concentrations as low as 4 μmol L^−1^ [65]. The synthetic peptide [Lys]^7^-Polybia-CP was also used in conjunction with micro- and nanomotors systems for the autonomous treatment of infections in an animal model [66]. These studies underscore the role of venom-derived peptides as scaffolds for novel antimicrobials.

### 2.5. Venom-Derived Peptides in the Clinic

From 2015 to 2020 the U.S. Food Drug Administration (FDA) approved 273 new drugs. Out of those, 21 were peptides or peptide-derived drugs described as medicines or drug delivery systems [67,68,69,70]. The sales of peptide drugs exceeded US$ 70 billion in 2019 with 10 non-insulin peptide drugs in the top 200 drug sales, representing a substantial part of the pharmaceutical market [71]. 

As noted above, biologically active peptides from venomous animals from South America display relevant and diverse activities and constitute promising clinical candidates. To date, some peptides from the cornucopia of venoms have been studied extensively based on their structure, activities, and toxicity. Captopril is a pioneering and successful example of the transition from venom to drug. This blockbuster angiotensin-converting enzyme (ACE) inhibitor was approved in 1981, under the name Capoten^®^ (Bristol-Myers Squibb, BMS, New York, NY, USA), for the treatment of cardiovascular diseases [72,73]. Captopril was designed based on bradykinin-potentiating peptides (BPP). Pro-rich peptides were found in the venom of the Brazilian pit viper *Bothrops jararaca* [2,72,73,74]. Just four years after its approval, Enalapril, another peptide produced by the same snake, was also approved under the name Vasotec^®^ (Merck, Darmstadt, Germany) for the treatment of hypertension and cardiac failure with higher therapeutic potency than Capoten^®^ but limited oral availability (Table 2) [72,75,76]. Batroxobin, found in the Brazilian lancehead snake *Bothrops moojeni* venom cocktail [72], is a thrombin-like serine protease that displays a high defibrinogenating effect with anti-inflammatory effects [72,77,78]. This activity allows the use of this peptide as a drug against thrombotic diseases (such as deep vein thrombosis, myocardial infarction, pulmonary embolism, and acute ischemic stroke), and as a diagnostic tool (Reptilase^®^) for the quantification of fibrinogen levels and blood coagulation capabilities (Table 2) [72,79]. This peptide has not yet been approved for clinical use in the USA by the FDA in the USA; however, it is commercialized in its original form under diverse brand names in other countries (Table 2), and it is currently in clinical phase IV for its use against cerebral venous sinus thrombosis [72]. The attractive activities displayed by venom-derived peptides enable their translation into preclinical development. Currently, ICK peptides from tarantulas are catching the attention of pharmaceutical companies for their ability to block Na_V_ channels and treat pain. For instance, the peptide PcTx-1 mentioned above in the spider venom section is currently undergoing preclinical trials, not only to assess its activity but also for its valuable role in furthering our understanding of ASIC_S_ heteromeric channels (Table 2) [72,80,81]. Recently, the ICK peptide protocin-II (ProTX-II) from the Peruvian tarantula *Thrixopelma pruriens* was optimized by Janssen via directed evolution achieving the novel peptide JNJ63955918, which exhibited improved selectivity for Na_V_1.7 channels along with in vivo tolerability (Table 2) [2,81,82]. The company Amgen has also explored this family of peptides and described and characterized the peptide GpTx-1, widely produced among South American tarantulas such as *Grammostila porter, Grammostila rosea*, and *Paraphysa scrofa* (Table 2). GpTx-1 is a peptide with high selectivity for the Na_V_1.7 channel (IC_50_ = 10 nM) that demonstrated to be an excellent template for the engineering of new bioactive peptides, such as the analog [Ala^5^, Phe^6^, Leu^26^, Arg^28^]GpTx-1 that demonstrated exceptional selectivity towards Na_V_1.7 (IC_50_ = 1.6 nM) and >1000 fold selectivity for Na_V_1.4 and Na_V_1.5 (Table 2) [2,81,83,84,85]. Of note, peptides can be administered within a mixture of active compounds to enhance the activity of a lead compound. This is the case with AMPs and CPPs, which primarily target the cell membrane, forming pores through diverse mechanisms of action that lead to cellular apoptosis. This characteristic not only allows their use as a single treatment but also the exploration of synergistic activities with different approved drugs. These peptides can act as adjuvants for compounds that have their target within the intracellular environment, allowing them to reach their active site efficiently, and increasing the effectiveness of the treatment by attacking the infection or illness through different mechanisms of action [86,87]. HYL, an α-helical antimicrobial peptide secreted within the venom of the solitary bee *Hylaeus signatus,* is an example of this synergistic activity. This peptide of poor antimicrobial activity was optimized following a structure−activity study that led to the generation of 25 new peptides (from HYL −1 to HYL-25) with optimized antimicrobial activities [88]. Combination of HYL and most of these analogs with rifampicin resulted in synergistic interactions (FIC ≤ 0.5) against *Pseudomona aeruginosa* [88]. This synergy was also observed when HYL-1, HYL-18, and HYL-25 were combined with tetracycline against the same bacterium [88]. Synergistic studies were also carried out against the Gram-positive bacterium *Staphylococcus aureus* by combination of the different HYL analogs with amoxicillin. Ten different analogs demonstrated FIC ≤ 0.5 against this bacterium [88]. 

## 3. Conclusions

Venom-derived peptides have demonstrated promising biological activities and potential as scaffolds for the generation of potent antimicrobials and anticancer agents through rational design. South American venomous organisms are important sources of biologically active peptides with antinociceptive, anticancer, and antibacterial properties. Recent advances in chemical and computational methods promise to accelerate the screening, testing, and lead identification of biologically active compounds.

## Figures and Tables

**Table 1:** Structural features and biological activities of venom-derived peptides from South American organisms. Extended overview of the number of amino acids (referred to as AA), sequence, peptide family, structure, biological activity, and producer organism of the venom-derived peptides. n.e. = not elucidated.

Peptide	AA	Sequence	Peptide Family	Structure	Activity	Organism Producer	Reference
**Snake Venom-Derived Peptides**							
Crotamine	42	YKQCHKKGGHCFPKEKICLPPSSDFGKMDCRWRWKCCKKGSG	β-defensin-like	β_1_αβ_2_β_3_ N-terminal 𝛼-helix, two stranded antiparallel β-sheets, and two β-turns.	Antifungal (MIC = 12.5–50.0 μg mL^−1^) Anticancer activity (5 μg mL^−1^)	*Crotalus durissus terrificus*	[25,34]
Crotalicidine	34	KRFKKFFKKVKKSVKKRLKKIFKKPMVIGVTIPF	Cathelicidin	α-helix at the N-terminal and random coil conformation at the C-terminal of the peptide.	Antibacterial (MIC < 10 μmol L^−1^), Anticancer (IC_50_ < 5 μmol L^−1^), Immunomodulatory activities	*Crotalus durissus terrificus*	[27,35]
**Spider Venom-Derived Peptides**							
PnTx1	78	AELTSCFPVGHECDGDASNCNCCGDDVYCGCGWGRWNCKCKVADQSYSYGICKDKVNCPNRHLWPAKVCKKCRRNCGG	ICK peptide	Disulfide bridge pattern	LD_50_ = 5.5 pmol g^−1^ (mice) Target: Nav channels, antagonist	*Phoneutria nigriventer*	[36,37]
PnTx2-1	53	ATCAGQDKPCKETCDCCGERGECVCALSYEGKYRCICRQGNFLIAWHKLASCK	ICK peptide	n.e.	Lethal in mice model at 0.02 pmol g^−1^	*Phoneutria nigriventer*	[36,37]
PnTx2-5	48	ATCAGQDQTCKVTCDCCGERGECVCGGPCICRQGNFLIAWYKLASCKK	ICK peptide	n.e.	Lethal in mice model at 2.4 pmol g^−1^	*Phoneutria nigriventer*	[36,37]
PnTx2-6	48	ATCAGQDQPCKETCDCCGERGECVCGGPCICRQGYFWIAWYKLANCKK	ICK peptide	n.e.	Lethal in mice model at 7.5 pmol g^−1^	*Phoneutria nigriventer*	[36,37]
PnTx2-9	32	SFCIPFKPCKSDENCCKKFKCKTTGIVKLCRW	ICK peptide	n.e.	-	*Phoneutria nigriventer*	[36,37]
PnTx3-1	41	AECAAVYERCGKGYKRCCEERPCKCNIVMDNCTCKKFISEL	ICK peptide	n.e.	Paralysis in mice at 0.07 pmol g^−1^ Target: agonist of K^+^ channels	*Phoneutria nigriventer*	[36,37]
PnTx3-2	46	ACAGLYKKCGKGASPCCEDRPCKCDLAMGNCICKKKFIEFFGGGK	ICK peptide	n.e.	Antagonist of L-type Ca_V_ channels. Paralysis in mice 0.08 pmol g^−1^	*Phoneutria nigriventer*	[36,37]
PnTx3-3	34	GCANAYKSCNGPHTCCWGYNGYLLACICSGXNWK	ICK peptide	n.e.	Lethal to mice at 0.07 pmol g^−1^ Antagonist of L-, P/Q- and R-type Cav channels	*Phoneutria nigriventer*	[36,37]
PnTx3-4	77	SCINVGDFCDGKKDDCQCCRDNAFCSCSVIFGYKTNCRCEVGTTATSYGICMAKHKCGRQTTCTKPCLSKRCKKNHG	ICK peptide	n.e.	Lethal to mice at 5 μg/mouse Target: antagonist of N-, P/Q- and R-type Ca_v_ channels	*Phoneutria nigriventer*	[36,37]
PnTx3-5	45	GCIGRNESCKFDRHGCCWPWSCSCWNKEGQPESDVWCECSLKIGK	ICK peptide	n.e.	Paralysis in mice 0.07 pmol g^−1^ Target: L-type Ca_v_ channels	*Phoneutria nigriventer*	[36,37]
PnTx3-6	54	ACIPRGEICTDDCECCGCDNQCYCPPGSSLGIFKCSCAHANKYFCNRKKEKCKK	ICK peptide	n.e.	Paralysis in mice 0.05 pmol g^−1^ Target: N-, P/Q- and L-type Cav channels	*Phoneutria nigriventer*	[36,37]
PnTx4-3	48	CGDINAACKEDCDCCGYTTACDCYWSSSCKCREAAIVIYTAPKKKLTC	ICK peptide	n.e.	Non-toxic to mice (288.5 pmol g^−1^) LD_50_ = 192.3 pmol g^−1^ (house fly)	*Phoneutria nigriventer*	[36,37]
PnTx4 (5-5)	47	CADINGACKSDCDCCGDSVTCDCYWSDSCKCRESNFKIGMAIRKKF-C	ICK peptide	n.e.	Non-toxic to mice (290 pmol g^−1^) Target: NMDAR (antagonist), Na_V_ channels (agonist)	*Phoneutria nigriventer*	[36,37]
PnTx4 (6-1)	48	CGDINAACKEDCDCCGYTTACDCYWSKSCKCREAAIVIYTAPKKKLTC	ICK peptide	n.e.	LD50=9.3 ng/house fly Non-toxic to mice (286.2 pmol g^−1^) ED_50_= 36.3 pmol g^−1^ (house fly) Target: agonist Na_V_ channels	*Phoneutria nigriventer*	[36,37]
Psalmotoxin 1 (PcTX1)	40	EDCIPKWKGCVNRHGDCCEGLECWKRRRSFEVCVPKTPKT	ICK peptide	Three antiparallel ®-sheet structure with three disulfide bridges tightly folded into the “knottin” fold pattern	Antinociceptive effects IC_50_ = 36 pmol L^−1^ in glioma cells.	*Psalmopoeus* *cambridgei*	[38,39]
U1-SCRTX-Lg1a	16	VGTDFSGNDDISDVQK	Anionic antimicrobial peptide (AAMP)	Random coil conformation with a <-helix structure between the ISDV residues	Active against Gram-negative bacteria (MIC 1.5–4.6 μmol L^−1^)	*Loxosceles* *gaucho*	[40]
**Scorpion Venom-Derived Peptides**							
Ts1	61	KEGYLMDHEGCKLSCFIRPSGYCGRECGIKKGSSGYCAWPACYCYGLPNWVKVWDRATNKC	®-like neurotoxin	Three antiparallel β-strands and a α-helix bonded by disulfide bridges	Toxic against mammals and insects Intravenous LD_50_ = 76 ± 9 μg kg^−1^ Target: Na^+^ channels	*Tityus serrulatus*	[41]
Ts2	62	KEGYAMDHEGCKFSCFIRPAGFCDGYCKTHLKASSGYCAWPACYCYGVPDHIKVWDYATNKC	®-like neurotoxin	three β-strands and one α-helix, and is arranged in a triangular shape forming a cysteine-stabilized α-helix/ β-sheet (CSab) motif. three β-strands and one α-helix, and is arranged in a triangular shape forming a cysteine-stabilized α-helix/ β-sheet (CSab) motif. three β-strands and one α-helix, and is arranged in a triangular shape forming a cysteine-stabilized α-helix/ β-sheet (CSab) motif three β-strands and one α-helix, and is arranged in a triangular shape forming a cysteine-stabilized α-helix/β-sheet (CSab) motif Cysteine-stabilized α-helix/β-sheet (CSαβ) motif composed of three β-strands and one α-helix arranged in a triangular shape	Induction of inflammation and production of cytokines Inhibition of the rapid inactivation of some Na_V_ channels	*Tityus serrulatus*	[41,42,43]
Ts3	62	KKDGYPVEYDNCAYICWNYDNAYCDKLCKDKKADSGYCYWVHILCYCYGLPDSEPTKTNGKC	α-neurotoxin	α-helix and three-stranded antiparallel β-sheet	Inhibition of the inactivation of Na_V_ channels Muscle relaxation	*Tityus serrulatus*	[41,44]
Ts5	64	KKDGYPVEGDNCAFACFGYDNAYCDKLCKDKKADDGYCVWSPDCYCYGLPEHILKEPTKTSGRC	α-neurotoxin	Core composed of three β-strands and one α-helix	LD_50_ = 94 ± 7 μg kg^−1^ in mice Causes hypertension Target: Na^+^ channels	*Tityus serrulatus*	[41,45]
Ts6	40	WCSTCLDLACGASRECYDPCFKAFGRAHGKCMNNKCRCYT	α-KTx (Potassium channel toxin)	α-helix and triple-stranded β-sheet stabilized by four disulfide bridges	Induction of inflammation and production of cytokines Blockage of K_V_ channels	*Tityus serrulatus*	[41,43]
Ts7	37	VFINAKCRGSPECLPKCKEAIGKAAGKCMNGKCKCYP	α-KTx (Potassium channel toxin)	n.e.	Blockage of K^+^ current Blockage of ^86^Rb efflux	*Tityus serrulatus*	[41]
Ts8	60	KLVALIPNDQLRSILKAVVHKVAKTQFGCPAYEGYCNDHCNDIERKDGECHGFKCKCAKD	®-KTx (Potassium channel toxin)	n.e.	Blockage of voltage-gated non-inactivating K^+^ channels from rat brain synaptosomes at IC_50_: 30 nmol L^−1^	*Tityus serrulatus*	[41]
Ts9	35	VVIGQRCYRSPDCYSACKKLVGKATGKCTNGRCDC	κ-KTx (Kappa potassium channel toxin)	Core composed of a short <-helix and a three-stranded antiparallel ®-sheet	Ligand for small-conductance apamin-sensitive calcium-activated potassium channel	*Tityus serrulatus*	[41]
**Wasp Venom-Derived Peptides**							
Polybia-MPI	14	IDWKKLLDAAKQIL	Mastoparan	71.43% α-helix	Antifungal activity (ED_50_ = 8–16 μmol L^−1^ Antimicrobial activity against Gram-positive and Gram-negative bacteria (MIC = 4–15 μg mL^−1^	*Polybia* *paulista*	[46,47]
Polybia-MPII	14	INWLKLGKMVIDAL	Mastoparan	α-helix	Antimicrobial activity against Gram-positive bacteria (MIC = 2–5 μmol L^−1^ Antifungal activity (ED_50_ = 111–12.9 μmol L^−1^ Hemolytic properties (ED_50_ of 5 × 10 ^-5^ mol L^−1^	*Pseudopolybia* *vespiceps Testacea, Polybia paulista*	[46]
Polybia-CP	12	ILGTILGLLKSL	Chemotactic peptide	50% random coil 50% ambiguous conformations	Antimicrobial activity against Gram-positive bacteria (MIC = 15 μg mL^−1^ Mast cell degranulation (10^−5^ mol L^−1^) Low hemolytic activity	*Polybia* *paulista*	[47]

**Table 2:** Clinical or preclinical status of venom-derived peptides from South America. Extended overview of the producer organism, description of the peptide, main biological target, target indication, company and brand, and clinical trial stage of the venom-derived peptides.

Peptide	Producer Organism	Description	Target	Target Indication	Company (Brand)	Clinical Trial	Reference
Captopril	*Bothrops* *jararaca*	Synthetic peptide based on bradykinin-potentiating peptides (BPP)	Angiotensin-converting enzyme (ACE)	Hypertension, cardiac failure	Bristol-Myers Squibb. (Capoten^®^)	Completed	[2,72,73,74,89]
Enalapril	*Bothrops* *jararaca*	Synthetic peptide based on bradykinin-potentiating peptides (BPP)	Angiotensin-converting enzyme (ACE)	hypertension and cardiac failure	Merck (Vasotec^®^)	Completed	[72,75,76,89]
Batroxobin	Brazilian lancehead snake (*Bothrops moojeni*)	Peptide isolated from venom	Cleavage of the Aα chain of fibrinogen at the [Ala]16-[Gly]17 bond	Defibrinogenating effect Anticoagulation therapy (thrombotic diseases) Diagnosis of fibrinogen levels and blood coagulation capabilities	Tobishi Pharmaceutical (Batroxobin, Reptilase, Defibrase) DSM Nutritional Products Ltd/Branch Pentapharm (Defibrase) Hanlim (Botropase) Juggat Pharma (Botropase, Botroclot) Drugs.com, (Botroclot) Plateltex S.R.O. (Plateltex-Act^®^) Vivostat A/S (Vivostat System).	Phase IV (Combination with anticoagulation in cerebral venous sinus thrombosis, NCT04269954) Completed in different countries	[72,77,78,79,90]
Psalmotoxin 1 (PcTx-1)	*Psalmopoeus* *cambridgei*	Peptide isolated from venom	Inhibitor of AISCs	Pain treatment	-	Preclinical	[72,80,81]
JNJ63955918	*Thrixopelma* *pruriens*	Synthetic peptide based on the natural peptide ProTX-II	NaV1.7 channels	Pain treatment	Janssen	Preclinical	[2,81,82]
GpTx-1	*Grammostila porter, Grammostila rosea*, and *Paraphysa scrofa*	Peptide isolated from the venom	NaV1.7 channels	Pain treatment	Amgen	Preclinical	[2,81,83,84,85]
[Ala5, Phe6, Leu26, Arg28]GpTx-1	*Grammostila porter, Grammostila rosea*, and *Paraphysa scrofa*	Synthetic peptide based on the natural peptide GpTx-1	NaV1.7, NaV1.5 and NaV1.4 channels	Pain treatment	Amgen	Preclinical	[2,81,83,84,85]

## Data Availability

Not applicable.
